# High Dose Inhalation with Gaseous Nitric Oxide in COVID-19 Treatment

**DOI:** 10.1134/S0006350922060185

**Published:** 2023-03-03

**Authors:** E. V. Pechyonkin, A. V. Kovrizhkin, A. V. Pekshev, A. B. Vagapov, N. A. Sharapov, A. F. Vanin

**Affiliations:** 1 Stavropol State Medical University, 355017 Stavropol, Russia; 2 City Clinical Hospital no. 3, 355029 Stavropol, Russia; 3 Bauman Moscow State Technical University, 105005 Moscow, Russia; 4 Semenov Federal Research Center for Chemical Physics Russian Academy of Sciences, 119334 Moscow, Russia

**Keywords:** nitric oxide, inhalation, dinitrosyl iron complexes, COVID-19, SARS-CoV-2, PLASON

## Abstract

A method of treatment of a new coronavirus infection COVID-19 in patients undergoing high flow oxygenation is proposed and technically implemented; the method is based on high-dose inhalation of gaseous nitric oxide (NO) with the patient’s spontaneous breathing. The results of the treatment of this disease demonstrating the high efficiency of the new method are presented. A possible mechanism of the blocking effect of high doses of inhaled nitric oxide on the replication of the SARS-CoV-2 virus is discussed; it is based on the formation of dinitrosyl iron complexes in the respiratory tract and lungs of COVID-19 patients with thiol-containing ligands acting as donors of NO and nitrosonium NO^+^ cations in a living organism that have a cytotoxic effect on the SARS CoV-2 virus.

## INTRODUCTION

The proposal on the possibility of using gaseous nitric oxide (NO) for the treatment of COVID-19 by inhalation into the patient’s respiratory system during spontaneous breathing appeared almost immediately after the outbreak of the pandemic [1].

The suggested mechanisms of action of inhaled NO in the treatment of COVID-19 are as follows: deactivation of the most important proteins of the SARS-CoV-2 virus necessary for its replication by S‑nitrosation of functionally important thiol groups in it [1, 2]; activation of T-lymphocytes, B-lymphocytes, NK cells, and macrophages responsible for the functioning of the cellular immunity system [3], especially in elderly patients with an age-related decrease in their production of endothelial nitric oxide [4] as well as a decrease in the likelihood of thrombosis due to the blocking effect of nitric oxide on platelet aggregation.

Data were presented on the treatment of COVID-19 patients with spontaneous respiration by inhalation of an air mixture containing 160 ppm of nitric oxide for 30 min twice a day [5, 6]. The very modest results of such treatment obtained in the United States can be explained by the data of our studies of the inhalation of gaseous nitric oxide by volunteers with NO content in the inhaled air in the range from 100 to 2100 ppm [2]. It was shown that, when the NO content in the inhaled air is less than 200 ppm, the vast majority of gaseous nitric oxide enters the blood through the lungs and binds to hemoglobin, as a result of which the remaining part of NO is insufficient to provide therapeutic effects. Based on the results of the studies in [2], we believe that the content of nitric oxide in the inhaled air should be at the level of approximately 1000 ppm in order to obtain a pronounced therapeutic effect. In this case, half of the nitric oxide absorbed by the body will enter the blood through the lungs and contact hemoglobin and half will remain in the respiratory system. The same opinion is held by the Nobel laureate, Professor L.J. Ignarro, who believes that the nitrogen oxide content in the NO-containing gas stream (NO-CGS) in the inhalation area should be at least 100 times greater than the NO content in known inhalation gas cylinder systems (approximately 10 ppm), that is, be at the level of approximately 1000 ppm [1].

## MATERIALS AND METHODS

### Technical Support

**Fig. 1.**
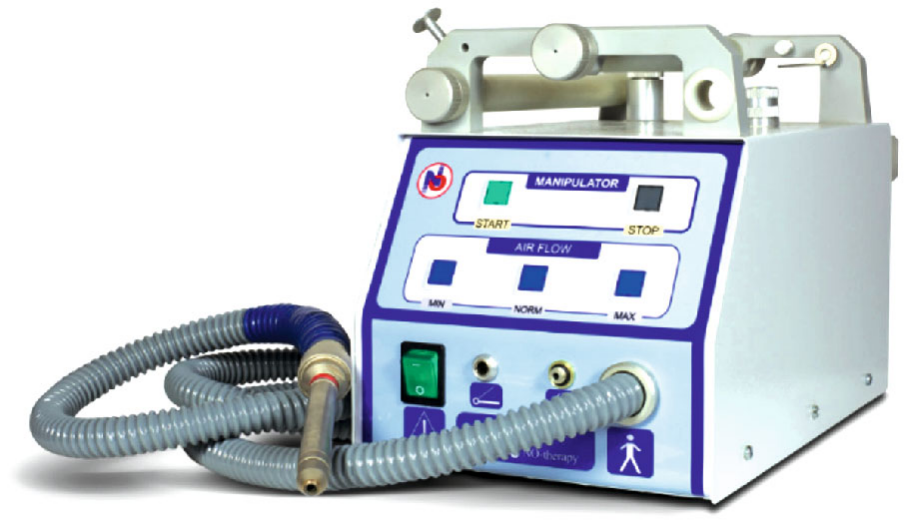
PLASON device.

The Scalpel-coagulator-stimulator air-plasma SKSVP/NO-01 PLASON device (TU 9444-001-96571701-2007), produced in 2012 by OOO TSVTM at Bauman Moscow State Technical University, Moscow, Russia (registration certificate no. FSR 2007/00583 dated March 21, 2012) was used in this study (Fig. 1).

The PLASON device is the only device in the world that implements a method of treating various diseases with an air stream containing nitric oxide obtained by plasma chemical method from atmospheric air (NO-therapy method).

The main application of the device is the treatment of wound pathology of various genesis, accelerating the healing of wounds, including long-term nonhealing postoperative, traumatic, purulent, burn, trophic ulcers, diabetic foot syndrome, bedsores, etc., and the treatment of erosive and ulcerative and inflammatory diseases of internal hollow organs (stomach, intestines, and lungs) by feeding nitric oxide on the pathology area through endoscopic devices.

For the possible use of the PLASON device for the treatment of COVID-19 patients, a special manipulator, a nitrogen oxide generator, was developed that provides safe high-dose inhalation of NO during spontaneous breathing of the patient.

When developing a manipulator for inhaled NO-therapy, we have widely used the experience gained in the development and application of plasma chemical generators of nitric oxide for experimental biomedical research presented in [7, 8] as well as data obtained by inhalation of healthy volunteers with a NO-containing gas stream [2].

To implement an effective and safe therapeutic technology of inhalation administration of nitric oxide into the respiratory system during spontaneous breathing, the following basic requirements were formulated.

1. For the comfort of patients and the ability to serve a large number of them sequentially with one device, the inhalation time should not exceed 10 min.

2. The content of nitric oxide in the NO-containing gas stream in the inhalation area should be at least approximately 1000 ppm.

3. The maximum amount of nitric oxide NO entering the respiratory system during inhalation, in accordance with the recommendations [9], should not exceed (in arb. un.) 12 000 ppm min (permissible dose of 25 ppm for 8 h).

4. The maximum amount of nitrogen dioxide NO_2_ entering the respiratory system during inhalation, in accordance with the recommendations [9], should not exceed (in arb. un.) 1440 ppm min (permissible dose of 3 ppm for 8 h).

5. It is not allowed to completely cool the NO-containing gas stream to room temperature, at which complete polymerization of nitrogen dioxide occurs with the formation of nitrogen tetraoxide (2NO_2_ = N_2_O_4_), which has a sharp unpleasant odor and is a highly toxic substance [8, 10].

6. After the end of the inhalation session, the concentration of methemoglobin formed in the blood when nitric oxide enters it and is its biological marker should not exceed 12% (in accordance with the work [11]) or 20% (in accordance with the work [12]).

**Fig. 2.**
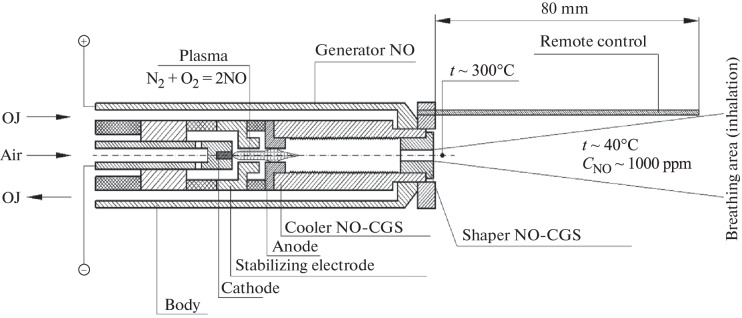
Scheme of plasma chemical generation of nitric oxide and formation of NO-CGS in an inhalation manipulator: coolant—coolant; NO-CGS—NO-containing gas stream.

Figure 2 shows a diagram of the developed manipulator for high-dose inhalation of nitric oxide, which is a plasma chemical generator of NO from atmospheric air and a shaper of NO-containing gas flow. The main elements of the manipulator design are a cathode (1 mm in diameter), a stabilizing electrode and an anode (with internal diameters of 1.1 and 1.5 mm, respectively) placed in a cylindrical housing (inner diameter of 12 mm), forcibly cooled by a liquid circulating in a closed cooling system of the manipulator. When the manipulator is operating, a low-current DC electric arc (approximately 2 A) is lit between the cathode and the anode, stabilized by the channel of the stabilizing electrode. Atmospheric air is supplied to the manipulator by a microcompressor, passes through an electric arc, and, passing into the plasma state, is heated to a high temperature sufficient for the effective synthesis of nitric oxide in accordance with the plasma chemical reaction N_2_ + O_2_ = 2NO. The high-temperature NO-containing gas stream obtained in this way flows through the cylindrical channel of the anode into the liquid-washed cooler integrated into the NO generator, passes through a cylindrical ribbed cooling chamber inside (length 35 mm, diameter 5 mm), and—through the output channel (diameter 1.1 mm) of the flow shaper—flows into the surrounding space in the form of a hot air stream containing two specific components, molecules of nitric oxide NO and nitrogen dioxide NO_2_, which formed as a result of the inevitable oxidation of a part of nitric oxide in an oxygen-containing medium: 2NO + O_2_ = 2NO_2_. The geometric parameters of the cooler provide a temperature of NO-CGS at the outlet of the flow shaper channel at approximately 300°C, at which the process of polymerization of nitrogen dioxide NO_2_ is prevented, leading to the formation of nitrogen tetraoxide N_2_O_4_, a toxic substance with a sharp unpleasant odor.

**Fig. 3.**
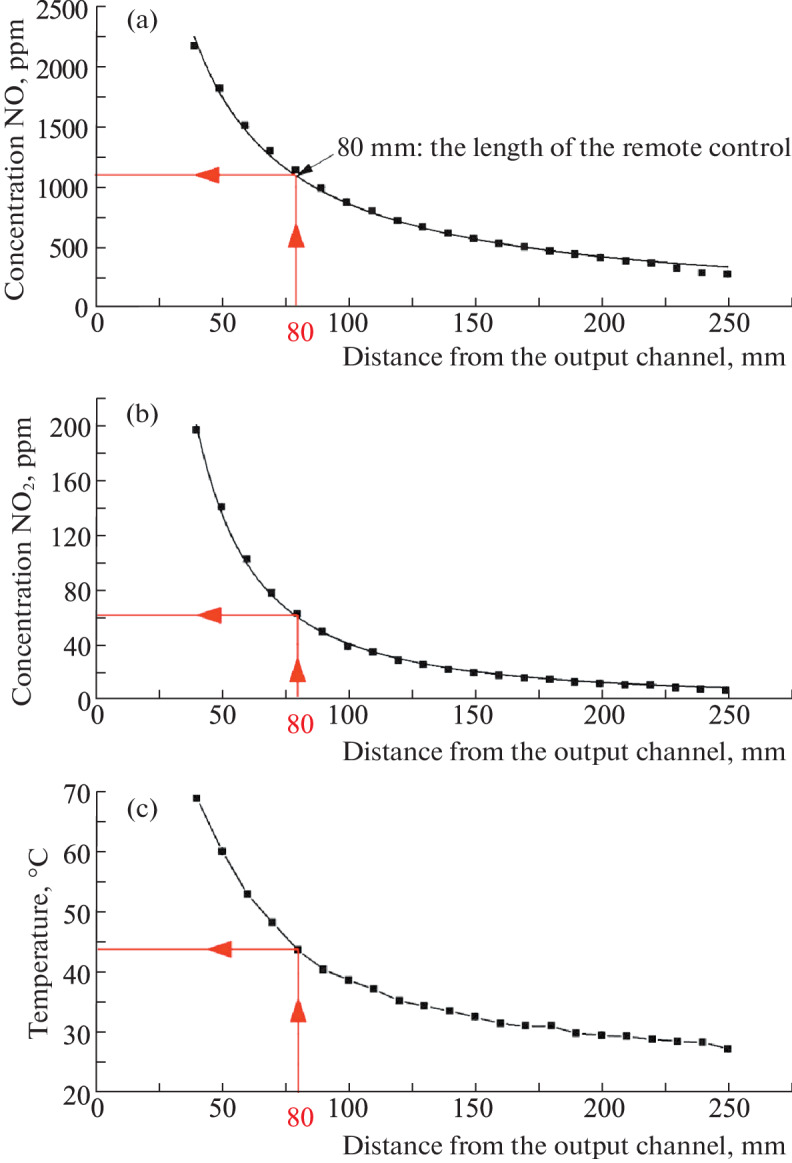
Basic physicochemical parameters on the axis of the NO-containing gas flow of the inhalation manipulator at an air flow rate of approximately 3 L/min.

Figure 3 shows the main physicochemical parameters on the axis of the NO-containing gas flow formed by the developed inhalation manipulator measured by the OPTIMA 7 gas analyzer manufactured by MRU GmbH (Germany). It can be seen that the NO-CGS region, suitable and acceptable for comfortable inhalation, is located at a distance of 80–90 mm from the output channel of the manipulator, where the flow temperature is 40–44°C, the content of nitric oxide is at the level of 1000–1100 ppm, while the content of nitrogen dioxide does not exceed 50–60 ppm, and the absence of an unpleasant sharp odor indicates the absence of nitrogen tetraoxide in the stream.

To fix the NO-CGS area suitable for inhalation, the manipulator is equipped with an 80 mm needle spacer; the amount of nitric oxide and nitrogen dioxide entering the respiratory system during a 10-min session is 11 000 ppm min and 600 ppm min, respectively, and does not exceed the values recommended in [9].

### Method Safety

**Fig. 4.**
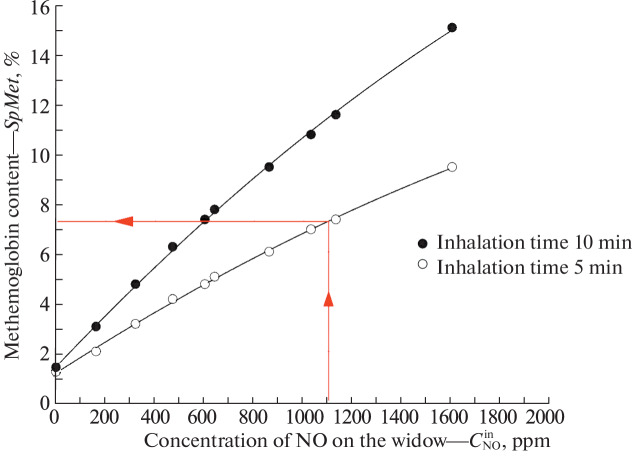
Content of methemoglobin in the blood of a healthy volunteer, a biological marker of nitric oxide after inhalation of gaseous NO.

Our studies on healthy volunteers have shown that inhalation of nitric oxide at its high content in the inhaled air (up to 2000 ppm) does not have a hypotensive effect under any circumstances and does not lead to a significant decrease in systemic blood pressure [2]. The developed manipulator was used on healthy volunteers and the content of methemoglobin in the blood was measured with a RAD-57 pulse oximeter manufactured by Masimo Corp. (United States) after nitric oxide inhalation in the range of changes in its content in the respiratory region from 160 to 1600 ppm with an inhalation duration of 5 and 10 min (Fig. 4). It was found that, with a nitric oxide content of approximately 1000 ppm in the stream, even after a 10 min session, the content of methemoglobin did not exceed the threshold 12%, which is safe for the patient.

**Fig. 5.**
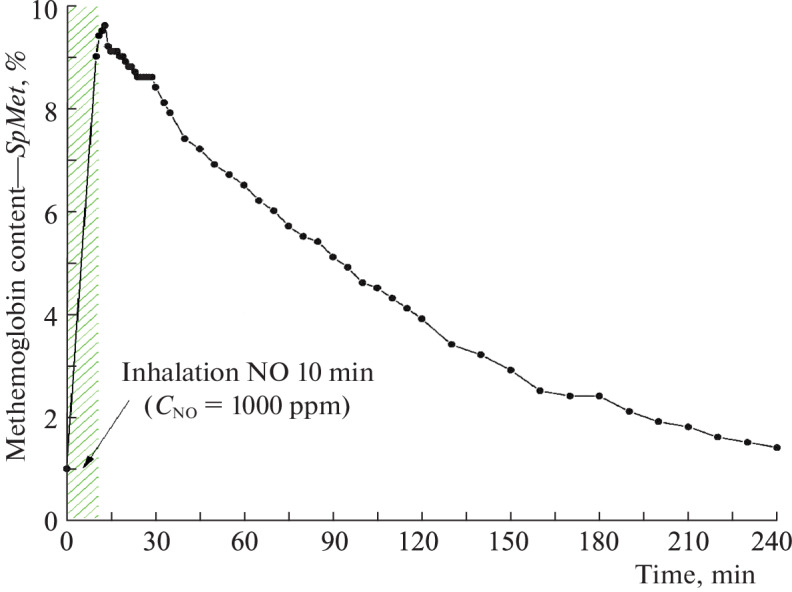
Dynamics of changes in the content of methemoglobin in the blood of a volunteer depending on the time after inhalation of nitric oxide.

Figure 5 shows the dynamics of changes in the content of methemoglobin in the blood depending on the time after inhalation of nitric oxide. It can be seen that the content of methemoglobin decreased to the physiological level within 3–4 h; the data were obtained using a pulse oximeter RAD-57.

The developed new manipulator for high-dose inhalation of nitric oxide is designed to work as part of the PLASON device, a detailed review of the clinical application of which is given in [13].

### Method of Using High-Dose NO Inhalation

The objective of the proposed new method of high-dose NO inhalation is to create an effective treatment of patients with a new coronavirus infection COVID-19 of moderate and high severity who are on high-flow oxygenation.

The proposed method of treatment consists in the fact that inhalation therapy with high concentrations of exogenous nitric oxide was carried out in addition to the standard protocol for the treatment of patients (etiotropic antiviral, anticoagulant, anti-inflammatory, antibacterial therapy) with pneumonia caused by COVID-19 of moderate and high severity, who are on high-flow oxygenation with preserved spontaneous breathing using a developed new manipulator as part of the PLASON device.

High-dose inhalation of nitric oxide was carried out in both nostrils of the patient with their spontaneous breathing from a distance of approximately 8 cm with a concentration of nitric oxide 1100 ppm for 5 min two to three times a day, alternating during the treatment session according to individual needs of inhalation of nitric oxide with high-flow oxygenation; in addition, the skin surface of the entire back of the patient in the supine position was treated with streams of nitric oxide from a distance of approximately 8 cm with a concentration of 1100 ppm for 5 min two to three times a day for 5–10 days before the relief of signs of acute respiratory distress syndrome.

The proposed new method of treating patients with COVID-19 with high-dose inhalation of NO is claimed by patent [14] and is applicable not only in inpatient intensive care units and intensive care units but also in conditions of mass admission of patients, is highly effective, safe, mobile, fully controlled, economically low-cost, and with minimal time spent on the procedure for one the patient. All patients note the clinical effect of subjective and objective improvement of respiratory functions immediately after the procedure. Immediately after the procedure, the level of blood saturation increases, respiratory function is restored, biochemical tests are improved, the psychological state improves, indications for intubation are excluded and critical-care physicians, gradually reducing the rate of high-flow oxygenation, transfer the patient to independent breathing.

## RESULTS

In total, treatment with a new method of high-dose inhalation of NO according to the method we proposed against the background of a standard treatment protocol in the covid intensive care unit of the Stavropol State Medical Institution “GKB no. 3” was carried out from October 2021 to February 2022 with the voluntary consent of 18 patients with COVID-19 with moderate and severe lung damage who were in extremely serious condition, with negative dynamics of treatment and an unfavorable prognosis, and who were in spontaneous respiration on high-flow oxygenation with an oxygen supply rate from 25 to 80 L/min. All patients avoided switching to artificial ventilation, survived, and recovered. No deaths were observed. Analyzing the results of clinical examples in the long-term period after rehabilitation therapy, it can be concluded that patients’ vital functions were fully restored. The new proposed method of treatment with high-dose NO inhalation is highly effective, capable of improving clinical outcomes with no restrictions on use; has optimal technical requirements and financial costs; contributes to rapid improvement of respiratory function, general condition, improvement of clinical results, and a significant reduction in deaths.

### Clinical Cases

### Example 1

Patient O., 69 years old. Diagnosis: new coronavirus infection COVID-19 of severe severity, community-acquired bilateral polysegmental pneumonia with the formation of CT-3-4 fibrosis (85% lung damage), pneumomediastinum, respiratory failure of the third degree, toxic anemia. Standard treatment was carried out according to the COVID-19 therapy protocol. The general condition is extremely serious. Against the background of progressively increasing respiratory insufficiency, the patient was transferred to high-flow oxygenation from 25 to 80 L/min, the content of C‑reactive protein (CRP) increased to 45 mg/L, pronounced coagulopathy. Due to the deterioration of the condition, it was planned to make a transfer to artificial ventilation of the lungs. The prognosis is considered unfavorable. With the voluntary consent of the patient, sessions of inhaled nitric oxide in high concentrations were conducted according to our proposed method: spontaneous breathing in both nostrils of the patient from a distance of 8 cm with a concentration of nitric oxide 1100 ppm for 5 min three times a day, alternating during the treatment session according to individual needs, inhalation of nitric oxide with high-flow oxygenation; in addition, the skin surface of the entire back of the patient in the supine position was treated with streams of nitric oxide from a distance of 8 cm with a concentration of 1100 ppm for 5 min three times a day for 8 days before the relief of signs of acute respiratory distress syndrome. The causal relationship of the procedure is clearly expressed and obvious, given the positive feedback from the patient himself and objective control of lung saturation. Saturation increased immediately during the session from 72 to 88%. After the first session, the patient noted an improvement in the respiratory function of the lungs, the pain when inhaling disappeared after 3 min of therapy, biochemical parameters improved during the day, CRP decreased from 45 to 15 mg/L per day, and coagulogram indicators normalized. The attending physician began to reduce the rate of high-flow oxygenation. Sputum discharge improved. The patient quickly began to recover against the background of regular procedures of inhalation of nitric oxide in high concentrations and exposure to NO in the lungs and back. Against the background of a smooth decrease in the rate of high-flow oxygenation under the control of normalizing saturation to 96%, he was transferred to independent breathing and he was discharged from the covid department with an improvement for rehabilitation therapy on the eighth day after the end of the course of NO exposure according to the method we proposed. The long-term health result was favorable.

### Example 2

Patient Ch., 62 years old. Diagnosis: new coronavirus infection COVID-19 of severe severity, community-acquired bilateral pneumonia of severe severity, respiratory failure of the third degree, CT-4 (80% lung damage), type 2 diabetes mellitus, insulin-dependent, unspecified erythrocytosis. Standard treatment was carried out according to the COVID-19 therapy protocol. The condition progressively worsened. The general condition is extremely serious. Against the background of progressively increasing respiratory insufficiency, the patient was transferred to high-flow oxygenation from 60 to 80 L/min, CRP increased to 28 mg/L, pronounced coagulopathy. Due to the deterioration of the condition, a transfer to artificial ventilation of the lungs was planned. The prognosis is considered unfavorable. With the voluntary consent of the patient, sessions of inhaled nitric oxide in high concentrations were conducted according to our proposed method: spontaneous breathing in both nostrils of the patient from a distance of 8 cm with a concentration of nitric oxide 1100 ppm for 5 min three times a day, alternating during the treatment session according to individual needs, inhalation of nitric oxide with high-flow oxygenation; in addition, the skin surface of the entire back of the patient in the supine position was treated with streams of nitric oxide from a distance of 8 cm with a concentration of 1100 ppm for 5 min three times a day for 10 days before the relief of signs of acute respiratory distress syndrome. Saturation increased immediately during the session from 75 to 90%. Immediately after the session, the patient noted the ease of breathing, the pain when inhaling disappeared after 3 min of therapy, the psychological state improved, biochemical parameters improved during the day, the CRP decreased from 28 to 5.4 mg/L in 2 days, and the coagulogram indicators normalized. The attending physician began to reduce the rate of high-flow oxygenation. Sputum discharge improved and cough intensity decreased. The patient quickly began to recover against the background of regular procedures of inhaled nitric oxide in high concentrations and through skin exposure to NO in the lungs and back. Against the background of a smooth decrease in the rate of high-flow oxygenation under the control of restored saturation of up to 98%, the patient was transferred to independent breathing. On the tenth day after the end of the course of exposure to NO according to the method we proposed, he was discharged from the covid department with an improvement for rehabilitation therapy. The long-term health result was favorable.

### Example 3

Patient D., 67 years old. Diagnosis: new coronavirus infection COVID-19 of severe severity, community-acquired bilateral bronchopneumonia of severe severity, CT-3-4 (70% lung damage), pneumomediastinum, respiratory failure of one to two degrees, coronary heart disease, atherosclerotic cardiosclerosis, hypertension of the third degree, risk four, NC IIA, FC2, diabetes mellitus two types first identified. Standard treatment was carried out according to the COVID-19 therapy protocol. The general condition is extremely serious. Against the background of progressively increasing respiratory insufficiency, the patient was transferred to high-flow oxygenation from 20 to 65 L/min, CRP increased to 52.4 mg/L; due to the deterioration of the condition, a transfer to artificial lung ventilation was planned. An unfavorable prognosis was possible. With the voluntary consent of the patient, sessions of inhaled nitric oxide in high concentrations were conducted according to the method we proposed: spontaneous breathing in both nostrils of the patient from a distance of 8 cm with a concentration of nitric oxide 1100 ppm for 5 min twice a day, alternating during the treatment session according to individual needs of inhalation of nitric oxide with high-flow oxygenation; in addition, the skin surface of the entire back of the patient in the supine position was treated with streams of nitric oxide from a distance of 8 cm with a concentration of 1100 ppm for 5 min twice a day for 5 days before the relief of signs of acute respiratory distress syndrome. Saturation increased immediately during the session from 76 to 92%. Immediately after the first session, the patient noted a significant improvement in the respiratory function of the lungs, the pain when inhaling disappeared, the psychological state improved, the patient had hope for a favorable outcome, biochemical parameters improved during the day, CRP decreased significantly (from 52.4 to 20.2 mg/L), coagulogram indicators normalized. The attending physician began to reduce the rate of high-flow oxygenation until cancellation. Sputum discharge improved, cough intensity decreased, appetite appeared. The patient quickly began to recover against the background of regular procedures of inhaled nitric oxide in high concentrations and exposure to NO in the lungs and back. Against the background of a smooth decrease in the rate of high-flow oxygenation under the control of normalizing saturation to 96%, she was transferred to independent breathing and she was discharged from the covid department with an improvement for rehabilitation therapy on the fifth day after the end of the course of NO exposure according to the method we proposed. The long-term health result is favorable.

### Example 4

Patient S., 77 years old. Diagnosis: new coronavirus infection COVID-19 of severe severity, ischemic stroke (lacunar subtype on the TOAST scale) in the blood supply zone of the left middle cerebral artery with the development of pronounced sensorimotor aphasia, right-sided prosoparesis, early recovery period, left-sided lower lobe pneumonia of moderate severity, respiratory failure of one to two degrees, ischemic heart disease, atherosclerotic cardiosclerosis, insufficiency mitral and aortic valves, tricuspid valve and pulmonary artery valve dysfunction, signs of atrial septal aneurysm, grade three hypertension, risk four, type two diabetes mellitus first identified, BCA atherosclerosis, bilateral tortuosity of both ICA with hemodynamic shift on the right up to 48%, mild iron deficiency anemia. Standard treatment was carried out according to the COVID-19 therapy protocol. The general condition is severe. Against the background of progressively increasing respiratory insufficiency, the patient was transferred to high-flow oxygenation from 20 to 35 L/min. An unfavorable prognosis was possible taking into account all concomitant diseases and age. With the voluntary consent of the patient, sessions of inhaled nitric oxide in high concentrations were conducted according to the method we proposed: spontaneous breathing in both nostrils of the patient from a distance of 8 cm with a concentration of nitric oxide 1100 ppm for 5 min twice a day, alternating during the treatment session, according to individual needs, inhalation of nitric oxide with high-flow oxygenation, while the skin surface of the entire back of the patient in the supine position was additionally treated with streams nitric oxide from a distance of 8 cm with a concentration of 1100 ppm for 5 min twice a day for 5 days before the relief of signs of acute respiratory distress syndrome. The causal relationship of the procedure is obvious given the positive feedback from the patient herself and the objective control of lung saturation. Saturation increased immediately during the session from 77 to 95%. Immediately after the first session, the patient noted a significant improvement in the respiratory function of the lungs, the pain when inhaling disappeared, the psychological state improved, the biochemical parameters improved during the day and coagulograms, the indicators of inflammation in the blood decreased significantly, cognitive and motor functions quickly began to recover. The attending physician began to reduce the rate of high-flow oxygenation until cancellation. Sputum discharge improved, cough intensity decreased. The patient quickly began to recover against the background of regular procedures of inhaled nitric oxide in high concentrations and exposure to NO in the lungs and back. Against the background of a smooth decrease in the rate of high-flow oxygenation under the control of normalizing saturation to 96%, she was transferred on the fourth day to independent breathing and after the end of the course of NO exposure, according to the method we proposed, she was transferred on the fifth day in satisfactory condition from the intensive care unit with improvement to rehabilitation therapy. In the future, she was safely discharged from the hospital and felt well. Taking into account the severity of the patient’s condition, concomitant diseases and age, to the great surprise of the attending physicians, the rapid recovery of the patient after inhalation NO-therapy according to the proposed method against the background of standard treatment seems highly effective and quite fast.

### Example 5

Patient M., 57 years old. Diagnosis: new coronavirus infection COVID-19 of severe severity, community-acquired bilateral polysegmental pneumonia of severe degree, CT-3-4 (70% lung damage), respiratory failure of the third degree. Standard treatment was carried out according to the COVID-19 therapy protocol. The condition progressively worsened. The general condition is extremely serious. Against the background of progressively increasing respiratory insufficiency, the patient was transferred to high-flow oxygenation from 25 to 60 L/min, biochemical parameters worsened. With the voluntary consent of the patient, sessions of inhaled nitric oxide in high concentrations were conducted according to our proposed method: spontaneous breathing in both nostrils of the patient from a distance of 8 cm with a concentration of nitric oxide 1100 ppm for 5 min three times a day, alternating during the treatment session according to individual needs, inhalation of nitric oxide with high-flow oxygenation; in addition, the skin surface of the entire back of the patient in the supine position was treated with streams of nitric oxide from a distance of 8 cm with a concentration of 1100 ppm for 5 min three times a day for 7 days before the relief of signs of acute respiratory distress syndrome. Saturation increased immediately during the session from 78 to 91%. Immediately after the session, the patient noted the ease of breathing, the pain when inhaling disappeared, the psychological state improved, the biochemical parameters of the blood improved during the day. The attending physician began to reduce the rate of high-flow oxygenation. Sputum discharge improved, cough intensity decreased. The patient quickly began to recover against the background of regular procedures of inhaled nitric oxide in high concentrations and through skin exposure to NO in the lungs and back. Against the background of a smooth decrease in the rate of high-flow oxygenation under the control of restored saturation, up to 95% was transferred to independent breathing. On the seventh day after the end of the course of exposure to NO according to the method we proposed, he was discharged from the covid department with an improvement for rehabilitation therapy. The long-term health result is favorable.

## DISCUSSION

As follows from the above, the use of high-dose inhalation of nitric oxide (at least 1000 ppm) in patients with varying degrees of complexity of COVID-19 disease has shown the high effectiveness of this approach in their treatment. All patients, even initially in extremely serious condition with an unfavorable prognosis, who were on high-flow oxygenation during spontaneous breathing (18 patients), survived and fully recovered, and without side post-cortical complications. As far as we know, such a result has not yet been obtained when using low-dose inhalation with gaseous NO (no more than 160 ppm) in the treatment of similar patients. The question arises: what could be the reason for the dramatic increase in the effectiveness of this method of treating patients infected with the SARS-Cov-2 coronavirus during the transition from low- to high-dose NO-inhalation of these patients. It is impossible to answer this question now. One can only suggest some possible mechanisms for this increase, which could consist of the following.

Firstly, when a large number of NO molecules entered the respiratory tract and lungs, a significant part of it could, when bound to superoxide anions, turn into highly cytotoxic peroxynitrite affecting the coronavirus. The implementation of this process could be facilitated by the high content in the respiratory tract and lungs of oxygen entering these tissues with air at atmospheric pressure. The subsequent decay of peroxynitrite at neutral (“physiological”) pH values, formed by the binding of superoxide and nitric oxide anions, should have led to the appearance in tissues of an extremely strong oxidizer, hydroxyl radical, which could block the replication of coronavirus. At the same time, this radical could, as a nonselective agent reacting with almost all intracellular components, thereby disrupt the flow of many metabolic processes and cause various pathological phenomena as a result. Since the latter were not detected in patients when high-dose NO-inhalation was applied to them, the mechanism of its therapeutic effect on patients with COVID-19 through peroxynitrite seems unlikely.

Secondly, with a high (atmospheric) oxygen content in the respiratory tract and lungs, NO could be oxidized to nitrogen dioxide (NO_2_), which binds to NO to form nitrogen trioxide (N_2_O_3_), capable as a donor of nitrosonium cation (NO^+^) *S*-nitrosate a variety of thiol-containing proteins, including those necessary for the implementation of covid infections, for example, host and virus proteases. However, the probability of S-nitrosating activity of nitrogen trioxide in cells could not be high enough due to the low stability of this compound due to its rapid hydrolysis at neutral (“physiological”) pH values to nitrite. In other words, nitrogen trioxide was not able to carry the hydrolyzable nitrosonium cation to thiol groups characterized by a higher affinity for nitrosonium cations compared to its affinity for hydroxyl anions in water molecules. Thus, this second mechanism of the inhibitory effect of gaseous NO on COVID-19 could hardly be largely probable.

Finally, the third mechanism of such influence may be determined by the ability of molecular nitric oxide to form so-called dinitrosyl iron complexes (DNIC) with thiol-containing ligands, both low-molecular and protein in animals and humans [15]. In accordance with modern ideas about these complexes, they could act in living organisms as both NO donors and nitrosonium cations [16]. At the same time, due to the sufficiently high stability of DNIC, especially DNIC of protein nature, these complexes could retain NO molecules and nitrosonium cations in the intracellular space as a kind of depot of these agents, selectively transferring them when colliding inside cells to the corresponding targets: heme- and thiol-containing proteins characterized by a higher affinity for these components of DNIC than these complexes themselves.

For the first time, the blocking effect of DNIC with thiol-containing ligands on viral infection was demonstrated by a group of German researchers who discovered the suppression of the activity of Coxsackie B virus protease 2A under the action of mononuclear dinitrosyl iron complexes (M-DNIC) with cysteine and, especially, M-DNIC, which included as a ligand the cysteine residue of the tetrapeptide Leu-Ser-Tre-Cis, selectively binding with protease A [17]. The blocking of this enzyme under the action of these complexes was detected both on an isolated protein and in experiments on the culture of myocardial cells infected with the Coxsackie B virus [17]. In both cases, the blockade was caused by reversible S-nitrosation of one of the thiol groups of protease A. This transformation in animal experiments led to a significant weakening of their infection with this virus [18].

The detected S-nitrosation of protease A under the action of DNIC with cysteine was obviously caused by nitrosonium cations released from DNIC. A similar conclusion about the mechanism of cytotoxic action on tumor cells of Jurkat M-DNIC with thiosulfate was later made in the work of a group of German and Russian researchers [19]. It turned out that this effect was not weakened but even enhanced by the combined action of M-DNIC with thiosulfate and one of the derivatives of dithiocarbamate on these cells: *N*‑methyl-D,L-glucamine dithiocarbamate.

**Scheme 1.**
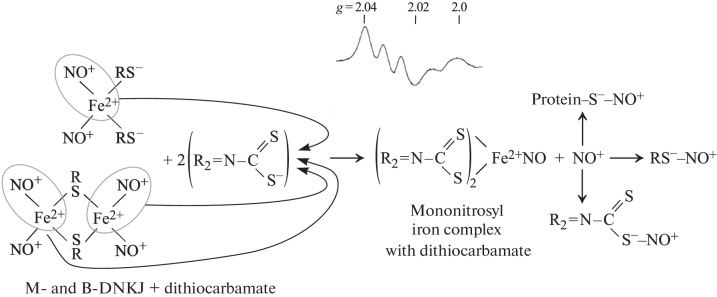
Mechanism of transformation of M- and B-DNICs with thiol-containing ligands into MNIC with dithiocarbamate derivatives. The nitrosonium cations released in this case can S-nitrosylate low-molecular and protein thiols as well as the thiol group in the composition of dithiocarbamate. The EPR signal registered at 77 K with *g*-factor values *g*_⊥_ = 2.04, *g*_||_ = 2.02 and a triplet hyperfine structure, characteristic of MNIC with dithiocarbamate, is shown at the top.

This increase was due to the ability of dithiocarbamate derivatives to pull over an iron-mononitrosyl (Fe^2+^-NO) group from an iron-dinitrosyl fragment of mononuclear and binuclear DNICs with the formation of stable biologically inactive mononitrosyl iron complexes with derivatives of dithiocarbamate and the release of nitrosonium cations from DNICs [20] (Scheme 1):

These cations were obviously responsible for cytotoxic effect of DNIC with thiosulfate on Jurkat cells.

This approach, namely, the use of a combination of binuclear DNICs with glutathione and a derivative of dithiocarbamate, diethyldithiocarbamate, during sequential (binuclear DNIC + diethyldithiocarbamate) aerosol treatment with solutions of these agents of Syrian hamsters infected with the SARS-CoV-2 coronavirus, made it possible to almost completely block this infection in animals [21].

In this regard, it is possible that the anticovid effect found in our work on patients with high-dose NO-inhalation was due to the formation of DNIC with thiol-containing ligands in the respiratory tract and lungs of these patients. The formation of these complexes could be facilitated by hypoxia of these organs, which occurs in them when they come into contact with a high amount of gaseous NO, the binding of which with oxygen led to hypoxia. The latter could increase the level of the DNIC component in the tissues of the respiratory tract and lungs, weakly bound (free) iron, which could ensure the appearance of a significant amount of DNIC in these tissues. The subsequent decay of these complexes, which ensures the accumulation of nitrosonium cations suppressing COVID-19 in tissues, could be initiated by superoxide anions that destroy DNIC.

This is the third of the above possible mechanisms of the anticovidic action of high (more than 1000 ppm) doses of gaseous NO—it seems most likely now. To prove this, only one thing is necessary: to check whether DNICs with thiol-containing ligands can occur in the body of animals and humans, with their high-dose NO-inhalation, in particular in the lungs.
